# Phenolic compounds, antioxidant, and antibacterial properties of pomace extracts from four Virginia‐grown grape varieties

**DOI:** 10.1002/fsn3.264

**Published:** 2015-08-07

**Authors:** Yixiang Xu, Sheanell Burton, Chyer Kim, Edward Sismour

**Affiliations:** ^1^ Agricultural Research StationVirginia State UniversityPetersburgVirginia23806; ^2^ Department of BiologyVirginia State UniversityPetersburgVirginia23806

**Keywords:** Antibacterial, antioxidant, extract, grape pomace, phenolic compounds, variety

## Abstract

Grape pomace is a potential source of natural antioxidant and antimicrobial agents. Phenolic compounds, antioxidant, and antibacterial properties of pomace extracts from four Virginia‐grown grape varieties were investigated. White grape pomaces had higher (*P* < 0.05) solvent extraction yield than red varieties. Concentrations of total phenolic (TPC), total flavonoid (TFC), total anthocyanin (TAC), tannins, condensed tannins (CT), as well as antioxidant capacities (DPPH• and ABTS•+free radical scavenging) differed (*P* < 0.05) among four pomace extracts. ABTS•+ scavenging capacity was positively correlated with TPC, TFC, tannins, and CT (*P* < 0.05), whereas DPPH• capacity was positively correlated with TAC (*P* < 0.05). Nine flavonoid compounds were identified, of which catechin and epicatechin were the two most abundant. Antibacterial activity was observed against *Listeria monocytogenes* ATCC 7644 and *Staphylococcus aureus* ATCC 29213, but not against *Escherichia coli* O157:H7 ATCC 3510 and *Salmonella typhimurium* ATCC 14028. *L. monocytogenes* was more susceptible than *S. aureus*.

## Introduction

Grape pomace refers to the solid remains following pressing of grapes for juice or winemaking, and consists primarily of the skin, pulp, seeds, and stems. Large quantities of grape pomace produced annually, and it has been reported that over 16 million tons of grape by‐products were produced in 2010 (González‐Centeno et al. 2013). Currently, grape pomace is used mainly for animal feed, organic fertilizers, ethanol production, or is direct disposed as a waste (Ben Rodn et al. 1994; Ferrer et al. 2001; Korkiel et al. 2002).

Grape pomace contains high level of polyphenols, and about 70% phenolic compounds were reported to remain in the pomace (Mazza 1995). Major phenolic compounds in the grape pomace are classified into two groups: flavonoid phenols (anthocyanins, flavanols, flavonols, and tannins) and nonflavonoid phenols (phenolic acids) (Ramirez‐Lopez and DeWitt 2014). Anthocyanins are pigments that are localized mainly in red grape skins, whereas flavonoids are localized in seeds and stems (Xia et al. 2010). The phenolic compounds in grape pomace extracts exhibit antioxidant, anticancer, and antidiabetic properties (Ruberto et al. 2007; Hogan et al. 2010; Parry et al. 2011; Zhou and Raffoul 2012; González‐Centeno et al. 2013), as well as antibacterial activity against *E. coli, L. monocytogenes,* and *S. aureus* (Ozkan et al. 2004a; Darra et al. 2012). The antioxidant activities exhibited by phenolic compounds are due to their free radical scavenging and metal chelating capacities that are influenced mainly by the number of OH− groups and their position in the phenol ring (Hogan et al. 2009). On the other hand, the antimicrobial activities of the phenolic compounds are attributed to an ability to bind extracellular and soluble proteins enabling complexation with bacterial cell walls (Puupponen‐Pimia et al. 2001). Because of these properties, polyphenols are being exploited to extend the shelf life of food products in response to increasing consumer concerns regarding synthetic preservatives (Gyawali and Ibrahim 2014), and to promote human health (Petti & Scully, 2009; Sagdic et al. 2011; Hasani and Hasani 2014; Teixeira et al. 2014).

It is worthy to note that there are varietal differences regarding the concentration and composition of phenolic compounds in grape pomaces and, consequently, in their resultant antioxidant and antimicrobial properties (Teixeira et al. 2014). Furthermore, environmental factors (e.g., geographical location, soil condition, and climate) and agronomic practices play key roles in influencing grape composition and associated properties. Most studies have focused on pomaces derived from red grape varieties, although some recent studies have examined white grape pomaces (González‐Centeno et al. 2013; Cerda‐Carrasco et al. 2015). Currently, no information is available concerning the pomaces from grape varieties produced in Virginia. Therefore, the objectives of the present study were to (1) quantify the composition and concentrations of phenolic compounds in pomaces from four selected Virginia‐grown grape varieties, and (2) assess their antioxidant and antibacterial activities to determine their potential as a source of natural antioxidants and antimicrobials.

## Materials and Methods

### Materials

Pomaces from four grape varieties widely used in Virginia for wine production were evaluated. Cabernet Franc and Chambourcin are red grape varieties, while Vidal Blanc and Viognier are white grape varieties. Cabernet Franc and Viognier are *Vitis vinifera* species, while Chambourcin and Vidal Blanc are hybrid grape variety. White grape pomaces are separated from the juice prior to winemaking, while red grape pomaces are separated after fermentation. Pomaces were obtained from two Virginia wineries. Chambourcin and Viognier were obtained from a winery in Goochland County, and Cabernet Franc and Vidal Blanc were obtained from a winery in Orange County. All chemicals and reagents were purchased from Fisher Scientific (Pittsburgh, PA) and Sigma‐Aldrich (St Louis, MO).

### Sample preparation

The pomaces were hand‐sorted to remove debris and stems, then freeze‐dried, and ground using a micro‐mill (IKA, Wilmington, NC) to pass through a size‐20 mesh sieve. Extracts were prepared by mixing 10 g of ground sample with 40 mL of aqueous acetone (80% v/v), followed by stirring for 24 h at room temperature, and then centrifugation at 10,000 ×  g for 20 min at 4°C. Supernatant was decanted into preweighed dishes and dried in a chemical hood with constant air flow. The dried extracts were weighed, resuspended in distilled water, and then filtered through 0.2 *μ*m syringe filters prior to conducting analyses.

### Total phenolic and tannins

The contents of total phenolic and tannis were determined using the Folin‐Ciocalteu method (Makkar et al. 1993) with some modifications. For total phenolic content (TPC), resuspended extracts were mixed with Folin–Ciocalteu reagent (10%) and sodium carbonate solution (7.5%), and then placed in the dark for 1 h, after which the absorbance was measured at 725 nm using a spectrophotometer (Evolution 60S, Thermo Scientific, Waltham, MA, USA). For simple phenolics, resuspended extract was first mixed with insoluble polyvinylpyrrolidone powder (100 mg) to adsorb tannins. The resulting supernatant was then reacted with Folin reagent as described as above. Tannin content was calculated as the difference between total and simple phenolics. Both TPC and tannins content were expressed as gallic acid equivalent (GAE) (mg/g sample) on a dry weight basis (dwb).

### Total flavonoid content

Total flavonoid content (TFC) was determined using the aluminum chloride assay described by Samatha et al. (2012). Briefly, resuspended extracts were mixed with sodium nitrite (5%). After standing for 6 min, aluminum trichloride (10%) was added and incubated for 5 min, followed by the addition of sodium hydroxide (4%) and distilled water. The absorbance was measured against a reagent blank at 510 nm using a spectrophotometer. TFC was expressed as catechin equivalents (CE) (mg/g sample, dwb).

### Total monomeric anthocyanins

Total monomeric anthocyanin content (TAC) was determined using the pH‐differential method described by Giusti and Wrolstad (2001). Resuspended extracts were diluted to the linear range of absorbance (less than 1.2) at 520 nm using potassium chloride buffer (0.025 m, pH 1.0) and sodium acetate buffer (0.4 M, pH 4.5). Absorbance was measured against a water blank at 520 nm (*λ*vis‐max) and 700 nm (to correct the haze) from 15 min to 1 h after sample preparation. TAC was expressed as cyaniding‐3‐glucoside equivalent (mg/g sample, dwb).

### Condensed tannins

Condensed tannins were measured according to Porter et al. (1986). Resuspended extracts were diluted with distilled water, followed by addition of butanol–HCl (95:5) and ferric reagents (2%). The mixtures were vortexed, held in a water bath (100°C) for 60 min, and then cooled to room temperature. Absorbance was measured at 550 nm. Condensed tannins concentration was expressed as leucocyanidin equivalent (% in dry matter).

### Antioxidant activity

DPPH• radical scavenging activity was determined according to Sánchez‐Moreno et al. (1998) with minor modifications. Briefly, resuspended extract (1.0 mL) was mixed with DPPH• solution (1.0 mL, 0.2 mmol/L, prepared daily) and stirred for 1 h at room temperature. Absorbance was measured at 515 nm. ABTS•+ radical scavenging activity was determined according to Re et al. (1999) with minor modifications. ABTS•+ reagent was prepared by incubating ABTS aqueous solution (7 mmol/L) with potassium persulfate (2.45 mmol/L), and was subsequently stored in the dark at room temperature for 12–16 h. Prior to use, the solution was further diluted to obtain an absorbance of 0.70 ± 0.02 at 734 nm. Resuspended extract (0.1 mL) was then mixed with ABTS•+ reagent (1 mL) for 1 min before measuring the absorbance at 734 nm. Both DPPH• and ABTS•+ radical scavenging activities were expressed as trolox equivalent (*μ*mol/g sample dwb).

### Identification of individual flavonoid compounds

Individual flavonoid compounds in the resuspended extracts were identified using HPLC (HP 1090, Agilent Technologies) with integrated a diode array detector. A gradient elution system having two mobile phases was used to separate individual compounds on a Synergy Hydro‐RP column (2.4 mm × 250 mm) (Phenomenex, Torrance, CA). Mobile phase “A” contained 100% acetonitrile and mobile phase “B” contained 3% acetic acid. Gradient elution was as follows: 5% A/95% B from 0 to 30 min; 25% A/75% B from 30 to 35 min; 75% A/25% B from 35 to 40 min (end of the run). The injection volume was 10 *μ*L, and the flow rate was 1.0 mL/min. The column temperature was set at 40°C. Absorbance of the elution was measured at 260 nm, 280 nm, 320 nm. Compounds were identified and quantified based on retention times and their peak areas were compared to those of known standards. Concentrations were expressed as mg/100 g grape extract.

### Antibacterial activity

#### Inhibition zones

Two species of pathogenic Gram‐positive bacteria (*Listeria monocytogenes* ATCC 7644 and *Staphylococcus aureus* ATCC 29213) and Gram‐negative bacteria (*Escherichia coli* O157:H7 ATCC 3510 and *Salmonella typhimurium* ATCC 14028) were used as test organisms. All bacterial cultures were grown separately in Mueller Hinton broth (MHB) for 22 ± 2 h at 36°C to obtain an inoculum concentration (~8 log CFU/mL). An agar‐well diffusion method was used to evaluate antibacterial activity of the pomace extracts (Ozkan et al. 2004b). In brief, the aliquots of each extract (100 *μ*L) at two concentrations (150 mg/mL and 300 mg/mL) were added to 7‐mm diameter wells cut into Mueller Hinton agar (MHA) plates infused with one of the four test organisms. Distilled water and chloramphenicol (5 mg/mL) were used as negative and positive controls, respectively. Plates were incubated at 36°C for 22 ± 2 h, after which, inhibition zones (mm) were measured to determine antibacterial activity.

#### Minimum inhibitory concentration and minimum bacteriocidal concentration

Minimum inhibitory concentration (MIC) and minimum bacteriocidal concentration (MBC) of the extracts were determined using a modified broth microdilution method (Nittiema et al. 2012). Resuspended extract (100 *μ*L) was transferred into the first well of a 96‐well sterile plate previously filled with MHB (100 *μ*L). Serial twofold dilutions were made by adding MHB and the bacterial inocula into consecutive wells. Test plates were incubated at 36°C for 22 ± 2 h. The serial dilutions were then overlain on plate count agar (PCA) and incubated at 36°C for an additional 22 ± 2 h prior to assaying for bacteriostatic and bacteriocidal activities. The lowest concentration of the extract exhibiting approximately same level of microbial growth as observed for the inoculated level was regarded as the MIC, while the lowest concentration of extract exhibiting no growth of bacteria was regarded as the MBC.

### Statistical analyses

Three replications of all assays were used to calculate means and standard deviations. Results were analyzed statistically using IBM^©^ SPSS^©^ Statistics, ver. 22, Armonk, NY: IBM Corp. One‐way ANOVA using the Duncan C post hoc test was used to evaluate the statistical significance of differences between grape varieties. Probability (*P*) ≤0.05 indicates statistical significance. Pearson's correlation coefficient (*r*) was used to evaluate covariance relationships between contents and antioxidant properties of pomace extracts.

## Results and Discussion

### Extraction yields and contents of phenolic compounds

Extraction yields differed significantly among grape varieties (Table 1). White grape pomaces had higher extraction yields than their red counterparts. This could be attributed to the different vinification processes for white and red grapes. The skins and seeds of white grapes are not fermented, therefore, most solvent extractable substances remain in the pomace. On the other hand, the skins and seeds of red grapes are fermented with the juice, so that extractable components wind up in the red wine. Additionally, extraction yield varied amonth the pomaces of both white and red grape varieties. Viognier pomace had significantly higher extraction yield than Vidal Blanc, and Cabernet Franc pomace showed a significantly higher yield compared to Chambourcin pomace.

**Table 1:** Total yield, phenolic, flavonoids and anthocyanins contents in the extracts from four grape pomaces

Varieties	Extraction yield (%)	Total phenolics (TPC) (mg GAE/g extract	Total flavonoids (TFC) (mg CE/g extract	Total anthocyanins (TAC) (mg Cyd‐3‐glu equivalent/g extract
Viognier	24.9 ± 1.00^a^	99.1 ± 0.29^b^	75.0 ± 0.42^b^	0.02 ± 0.01^c^
Vidal Blanc	20.6 ± 2.57^b^	55.5 ± 0.87^d^	32.8 ± 0.41^d^	0.06 ± 0.01^c^
Cabernet Franc	12.4 ± 0.55^c^	153.8 ± 1.83^a^	91.7 ± 1.00^a^	1.38 ± 0.03^b^
Chambourcin	5.30 ± 0.61^d^	92.0 ± 2.16^c^	38.9 ± 0.74^c^	10.7 ± 0.05^a^

GAE, gallic acid equivalent; CE, catechin equivalent.

Data are expressed as mean ± standard deviation (*n* = 3).

Means followed by the same letter within a column indicate no significant (*P* > 0.05) difference among samples.

Total phenolic (TPC) and total flavonoid (TFC) differed significantly, while exhibiting the same rank order among the four pomace extracts (Table 1). The extract from Cabernet Franc pomace had the highest TPC and TFC, followed by those from Vioginer, Chambourcin, and Vidal Blanc pomaces. Cabernet Franc pomace extract examined in the present study had higher TPC (153.8 mg GAE/g) and TFC (91.7 mg CE/g), compared to those reported by Hogan et al. (2010), TPC of 30.4 mg GAE/g and TFC of 22.1 mg RE/g. The differences could arise from variations in genetic backgrounds, environmental factors, agronomic practices, or vinification processes (Doshi et al. 2006). Furthermore, Yang et al. (2009) reported fresh Cabernet Franc grape extracts had TPC and TFC of 4.2 mg GAE/g and 1.8 mg CE/g, and fresh Vidal Blanc grape extracts had TPC and TFC of 2.3 mg GAE/g and 1.0 mg CE/g. Significant differences in TPC and TFC between pomaces and fresh grapes are attributed to the localization of phenolic compounds mainly in the skin and seeds of grapes.

Total monomeric anthocyanins (TAC) in the four pomace extracts differed significantly (Table 1). As expected, the red grape pomaces had significantly higher TAC than their white counterparts, since anthocyanins are a major pigment for red, purple, and blue colors, and are present exclusively in the skin of red grapes (He et al. 2012). Of the two red grapes examined, TAC content in the Chambourcin pomace extract was approximately eightfold higher than in the Cabernet Franc. Our results for red grape pomaces are in the agreement with Rockenbach et al. (2011) who reported TAC of four red grape pomaces ranging from 1.84 to 11.2 mg cyaniding‐3‐glucoside equivalent/g.

Tannin content followed the same trend as TPC, and was the highest for Cabernet Franc pomace extract, followed by Viognier and Chambourcin, and was the lowest for Vidal Blanc (Table 2). Tannins are localized primarily in the skin and seeds of grapes, and can be divided into condensed tannins and hydrolyzable tannins (Alipour and Rouzbehan 2010). Condensed tannins (CT), also known as proanthocyanidins, are highly insoluble and consist of flavan‐3‐ols monomers subunits, and contribute to bitter and astringent tastes of grapes and wines (Fontoin et al., 2008). Four pomace extracts had CT ranging from 8.61% to 50.5% leucocyanidin equivalent, which is in the agreement with Rondeaua et al. (2013) who reported CT value of 21% to 52% for pomaces from French vineyard grapes. Two red grape pomace extracts had higher CT compared to their two white counterparts. Moreover, CT was significantly higher in the extract of Cabernet Franc pomace compared to that of Chambourcin, while Viognier pomace extract had higher CT compared to that of Vidal Blanc.

**Table 2:** Tannins, condensed tannins and antioxidant activities in the extracts from four grape pomaces

Varieties	Tannins (mg GAE/g extract)	Condensed Tannins (% leucocyanidin equivalent)	DPPH (µmol TE/g extract)	ABTS (µmol TE/g extract)
Viognier	98.6 ± 0.17^b^	25.7 ± 2.56^c^	3.54 ± 0.06^a^	951 ± 44.4^b^
Vidal Blanc	54.5 ± 0.85^d^	8.61 ± 0.95^d^	7.71 ± 0.02^b^	334 ± 7.39^d^
Cabernet Franc	152.2 ± 2.56^a^	50.5 ± 0.19^a^	11.2 ± 0.17^c^	1013 ± 77.3^a^
Chambourcin	88.5 ± 2.40^c^	36.3 ± 1.52^b^	28.2 ± 0.02^d^	378 ± 11.8^c^

GAE, gallic acid equivalent; TE, trolox equivalent.

Data are expressed as mean ± standard deviation (*n* = 3).

Means followed by the same letter within a column indicate no significant (*P* > 0.05) difference among samples.

### Antioxidant activities

Free radical scavenging capacities differed significantly among the four pomace extracts, and diffrened between the DPPH• and ABTS•+ assay systems (Table 2). DPPH• scavenging capacity ranged from 3.54 to 28.2 µmol TE/g among the four pomace extracts, with the highest scavenging capacity exhibited by Chambourcin, followed by Cabernet Franc, Vidal Blanc, and Viognier. Hogan et al. (2009) investigated antioxidant activities of two fresh Virginia‐grown Cabernet Franc grapes and found their DPPH• scavenging activities were 5.4 and 8.8 µmol TE/g. The pomace extracts examined in the present study also exhibited significant ABTS•+ scavenging capacity ranging from 334 to 1013 µmol TE/g, with the order of Cabernet Franc > Viognier > Chambourcin >Vidal Branc. ABTS•+ scavenging capacity in the present study are higher than those reported previously for red grape pomaces (193–485 µmol TE/g) and for white grape pomaces (71–134 µmol TE/g) (Rockenbach et al. 2011; González‐Centeno et al. 2013).

### Relationship between phenolic compounds and antioxidant activity

TPC, TFC, tannins, CT exhibited significant, positive correlations with each other, as well as with ABTS •+ scavenging capacity (Table 3). However, these compounds and ABTS•+ scavenging activity were uncorrelated with either TAC or DPPH• scavenging activity, though TAC and DPPH• exhibited significant, positive correlation with each other. These results suggest that different phenolic compounds are responsible for quenching different free radicals. Flavonoids, tannins, and condensed tannins contribute to ABTS•+ antioxidant capacity, while anthocyanins contribute to DPPH• antioxidant capacity. Elfalleh et al. (2012) found negative correlations between TPC, TFC, TAC, and hydrolyzable tannins with DPPH• scavenging capacity, and no correlation between these phenolic compounds with ABTS •+ scavenging capacity in pomegranate juice. The distinctive structure of each phenolic compound (number of OH groups, side chain on benzoic acid) explains their special capacities to scavenge different free radicals (Tabart et al. 2009).

**Table 3:** Correlation coefficients between the contents of phenolics and antioxidant activity

	TPC	TFC	TAC	Tannins	CT	DPPH	ABTS
TPC	1.000	0.889***	0.132	0.999***	0.931***	0.018	0.798**
TFC			−0.292	0.902***	0.686*	−0.409	0.977***
TAC		1.000	1.000	0.101	0.330	0.991***	−0.386
Tannis				1.000	0.919***	−0.013	0.815**
CT					1.000	0.369	0.577*
DPPH						1.000	−0.501
ABTS							1.000

Significant correlations are indicated as: *(0.05 >  *P* > 0.01), ** (0.01 >  *P* > 0.001), *** (0.001 > *P*).

### Individual flavonoid compounds

**Figure 1:**
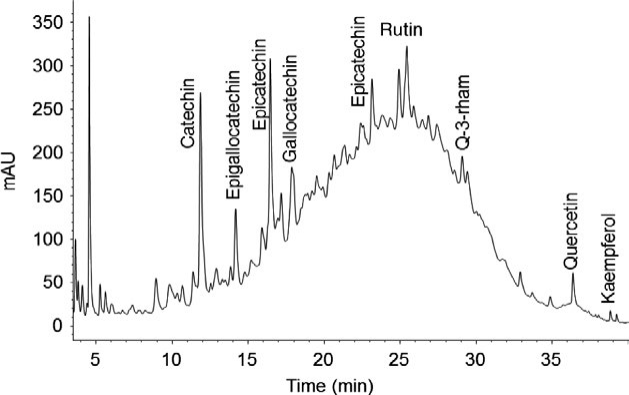
A typical HPLC chromatogram of grape pomace extract at 280 nm.

Nine flavonoid compounds, catechin, epicatechin, epigallocatechin gallate (EGCG), gallocatechin gallate (GCG), epicatechingallate (ECG), quercetin, quercetin‐3‐rhamnoside, kaempferol, and rutin were identified using HPLC (Fig. 1), and their concentrations are summarized in Table 4. Catechin, epicatechin, quercetin, and rutin were found in all four extracts, though their concentrations differed significantly. Catechin was the most abundant flavonoid compound. This agrees with previous report on phenolic compounds in grape pomace (Rockenbach et al. 2011). Further, white grape pomace extracts had higher catechin and epicatechin concentrations compared to the red ones. This is consistent with the findings of Nile et al. (2013). Catechin and epicatechin were the highest in Viognier pomace extract, and the lowest in Chambourcin pomace extract. Rutin content in the four extracts was in the order of Vidal Blanc > Cabernet Franc > Viognier > Chambourcin. The extracts from red varieties had the highest quercetin contents. Kaempferol was found only in the red varieties, whereas quercetin‐3‐rhamnoside was found only in the white varieties. Vuorinen et al. (2000) investigated flavonol contents of different types of grape wines and did not detect quercetin or kaempferol in white wines. Of three catechin and gallic acid esters, EGCG was highest in Cabernet Franc, followed by Viognier and Vidal Blanc, but was not quantified in Chambourcin. Cabernet Franc had the highest GCG followed by Chambourcin and Viognier, whereas Viognier had the highest ECG, GCG, and ECG were not detected in Vidal Blanc.

**Table 4:** Major individual flavonoid compounds in the extracts from four grape pomaces

Flavonoid compounds (mg/g extract)	Viognier	Vidal Blanc	Cabernet Franc	Chambourcin
Catechin	910 ± 10.5^a^	631 ± 13.4^b^	560 ± 4537^b^	214 ± 4.80^c^
Epicatechin	625 ± 9.20^a^	451 ± 22.2^b^	215 ± 4.67^c^	109 ± 4.17^d^
Epigallocatechin gallate	96.1 ± 3.47^b^	62.8 ± 0.78^b^	171 ± 7.26^a^	–
Gallocatechin gallate	99.1 ± 1.29^c^	–	232 ± 3.19^a^	146 ± 3.37^b^
Epicatechingallate	427 ± 11.7^a^	–	122 ± 2.995^b^	56.9 ± 5.36^c^
Quercetin‐3‐rhamnoside	27.1 ± 2.59^a^	33.5 ± 1.57^a^	–	–
Quercetin	17.3 ± 0.38^c^	20.7 ± 0.01^c^	56.5 ± 1.95^a^	31.2 ± 2.26^b^
Kaempferol	–	–	8.69 ± 0.38^a^	3.28 ± 0.14^b^
Rutin	255 ± 16.7^c^	435 ± 14.0^a^	343 ± 11.0^b^	99.5 ± 0.39^d^

Data are expressed as mean ± standard deviation (*n* = 2).

Means followed by the same letter within a row indicate no significant (*P* > 0.05) difference among samples.

–, stands for not detectable.

### Antibacterial activity

**Figure 2:**
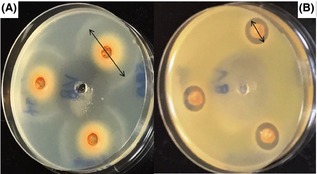
Antibacterial activity (inhibition zone) of Vidal Blanc pomace extract against (A) *L. monocytogenes* ATCC 7644 and (B) *S. aureus* ATCC 29213

The antibacterial activity of four grape pomace extracts is presented in terms of size of inhibition zone (mm) (Table 5). All extracts exhibited antibacterial activity against *L. monocytogenes* and *S. aureus* (Fig. 2), but no antibacterial activity was dected against *E. coli* O157:H7 and S. typhimurium. Our results agree partially with previous studies of whole grape or grape pomace extracts that found antibacterial activity against both Gram‐positive and Gram‐negative bacteria, and that extracts were more effective against Gram‐positive bacteria (Darra et al. 2012; Oliveira et al. 2013). The difference between our results and these others could be associated with different interpretations of the inhibition zone. The unique cell structure (two layer cell membrane and strong hydrophilicity of the outer membranres) of the Gram‐negative bacteria explains their strong resistance to pomace extracts (Smith‐Palmer et al. 1998).

**Table 5:** Antibacterial activity of the extracts from four grape pomaces

Varieties	Conc. (mg/mL)	Diameters of inhibition zone (mm)			
		Gram‐positive		Gram‐negative	
		*Listeria monocytogenes* ATCC 7644	*Staphylococcus aureus* ATCC 29213	*Escherichia coli* O157:H7 ATCC 3510	*Salmonella Typhimurium* ATCC 14028
Viognier	150	23.6 ± 0.6^a^	7.8 ± 0.5^b^	Nd	Nd
	300	25.0 ± 0.6^a^	8.6 ± 0.4^b^	Nd	Nd
Vidal Blanc	150	21.2 ± 0.9^b^	1.2 ± 0.0^e^	Nd	Nd
	300	25.1 ± 0.4^a^	2.9 ± 0.6^d^	Nd	Nd
Cabernet Franc	150	16.4 ± 1.0^c^	6.9 ± 0.4^c^	Nd	Nd
	300	21.6 ± 0.6^b^	11.7 ± 0.3^a^	Nd	Nd
Chambourcin	150	14.0 ± 1.0^c^	5.7 ± 0.1^c^	Nd	Nd
	300	N/A	N/A	Nd	Nd

N/A, not available; Nd, not detectable.

Data are expressed as mean ± standard deviation (*n* = 3).

Means followed by the same letter within a column indicate no significant (*P* > 0.05) difference among samples.

Among the Gram‐positive strains, *L. monocytogenes* was more susceptible than *S. aureus*, but susceptibility was influenced by grape variety and extract concentration. At an extract concentration of 150 mg/mL, the susceptibility of *L. monocytogenes* was the highest for Viognier, followed by Vidal Blanc, Cabernet Franc, and Chambourcin. Inhibition increased significantly with increasing extract concentration to 300 mg/mL for Vidal Blanc and Cabernet Franc, but not for Viognier. In comparison, at an extract concentration of 150 mg/mL, Viognier had the highest inhibition activity against *S. aureus*, followed by Cabernet Franc, Chambourcin, and Vidal Blanc. As with *L. monocytogenes*, inhibition of *S. aureus* increased significantly with increasing concentration to 300 mg/mL for Vidal Blanc and Cabernet Franc, but not for Viognier.

The agar diffusion method is considered as a qualitative test for initial screening of the antibacterial activity of a substance to provide indication for further quantitative evaluation of minimum inhibition (MIC) and minimum bactericidal concentration (MBC) (Oliveira et al. 2013). MIC and MBC of our four pomace extracts against *L. monocytogenes* ranged from 4.69 to 18.8 mg/mL and from 9.38 to 37.5 mg/mL, respectively (Table 6). Cabernet Franc pomace extract showed the lowest MIC (4.69 mg/mL) and MBC (9.38 mg/mL). On the other hand, higher MIC (40.6 to 250 mg/mL) was observed for the extracts against *S. aureus* ATCC 29213, and this strain even survived while extract concentration excedding 250 mg/mL for Vidal Blanc pomace. The MIC and MBC results indicated that the grape pomace extracts had higher antibacterial activity against *L. monocytogenes* than *S. aureus*, which are consistent with the results obtained from agar‐well diffusion method.

**Table 6:** Minimum inhibitory concentration (MIC) and minimum bactericidal concentration (MBC) of four grape extracts against *Listeria monocytogenes* and *Staphylococcus aureus*

Varieties	*Listeria monocytogenes* ATCC 7644		*Staphylococcus aureus* ATCC 29213	
	MIC (mg/mL)	MBC (mg/mL)	MIC (mg/mL)	MBC (mg/mL)
Viognier	5.07^a^	10.2^a^	40.6^a^	162.5^b^
Vidal Blanc	15.6^b^	31.3^b^	250^c^	>250
Cabernet Franc	4.69^a^	9.38^a^	75^b^	150^b^
Chambourcin	18.8^b^	37.5^b^	75^b^	75^a^

Data shown for MICs and MBCs are a result of four replicates of which three values were identical for every organism and every tested sample**.**

Means followed by the same letter within a column indicate no significant (*P* > 0.05) difference among the samples.

## Conclusion

Significant differences were found among the pomaces from four Virginia‐grown grape varieties in relation to the concentrations of total phenolics, total flavonoids, total anthocyanins, tannins, and condensed tannins and to DPPH• and ABTS•+ free radical scavenging assays. Cabernet Franc pomace extract exhibited the highest content of phenolics compounds and ABTS•+ free radical scavenging activity. Total phenolics, total flavonoids, tannins, and condensed tannins were positively correlated with each other and with ABTS•+ scavenging capacity. There was a positive correlation between total anthocyanins and DPPH• ‐scavenging capacity. All pomace extracts exhibited antibacterial activity against *L. monocytogenes* ATCC 7644 and *S. aureus* ATCC 29213, but not against *E. coli* O157:H7 ATCC 3510 or *S. typhimurium* ATCC 14028. Cabernet Franc and Viognier pomaces appear to have the great potential as source of natural antioxidant and antimicrobial agents. Further research will focus on further identification of phenolic compounds, and the relationship of individual phenolic compounds to antioxidant and antibacterial activities in grape pomaces.

## Conflict of Interest

None declared.
